# Calomel in the Treatment of Cholera

**Published:** 1849-06

**Authors:** 


*Calomel in the Treatment of Cholera.*—The Provincial Medical and Surgical Journal has the following on the treatment of cholera.

Mr. Stedman arrived off Calcutta in the early part of October, 1817, a few weeks after the cholera first broke out at Jessore. The ship, on board of which he was the appointed surgeon, was eleven days kedging up the Hoogly river, and, as each day's progress lessened the distance to the capital, so it increased the horrors of the spectacle presented by the numerous dead bodies floating, up and down, with the flux and reflux of the tides. Mr. Stedman was the first person in the ship who was attacked by the cholera. probably, as was strongly impressed on his mind at the time, because, as a professional man, he took more interest, than any other individual on board, in noticing the numbers, forms, colors, etc., of the dead bodies, and, particularly looking on, when any native was performing his duty, as police, in moving such of them as happened to get entangled, when the ship as at anchor, at the bows, by the cable, or otherwise. The Bengal papers of that period stated, that between two and three thousand dead were cast into the river in a week. Hence Mr. Stedman's exposure to a sufficient force of putrefying animal effluvia to account for his being the first individual in the ship seized by the disease.

His plan of treatment was simple and bold, but most unequivocally successful, since he lost not one of the crew, amounting to forty-eight, all of whom, with only one single exception, had the disease, whilst other ships in the river lost many of their men. A Bristol ship, moored about two cable lengths higher up the stream, lost thirteen of her crew the first fortnight after her arrival.

Mr. Stedman's sheet-anchor and sole reliance was calomel. Having commenced dosing himself, and having repeated it to a successful issue, he followed the same plan in all the other cases as they occurred. He was, as it were, knocked down by the first seizure, and instantly rendered unable to go to the medicine chest. He requested the chief officer to go and weigh twenty grains of calomel for him. He replied that he would bring the calomel and the scales, but that he was not going to give such a dose as that. By the time he returned into the cabin, Mr. Stedman had incessant vomiting, but no power. He begged the officer to weigh twenty grains, and then to slip it off the scale upon his tongue. This done the vomiting ceased for a short time, but not the cramps in the abdomen or limbs. On the recurrence of the retching, Mr. Stedman again, in less than ten minutes, requested to have twenty grains more. This checked all further vomiting, and, in one hour afterwards, still having spasmodic pains and drawings of the abdominal muscles, with heavings to vomit, he asked for twenty grains more. This, the first officer refused to have anything to do with, but the second officer and others standing around overruled the objection, and a third dose of twenty grains was given. Shortly after this, Mr. Stedman was so far relieved as to be able to sit upright in the chair, and then they made him sip brandy and water. This warmed his stomach, and brought on reaction of the heart, and, although very feeble, with frequent cold-sweats for several days, he might be said to have been well next day, as he was able to walk about, and attend to his duty.

The second case occurred the third day after Mr. Stedman's attack; and very rapidly all on board, with the single exception already mentioned, of the chief officer, (who entirely escaped,) were seized. The captain who had gone ashore, and remained there the whole time, also escaped.

To some of the men Mr. Stedman found it necessary to give to the extent of five doses, and, to one individual, six doses of twenty grains each. In two instances he began with half-drachm doses. In all the cases this simple plan proved successful. The only adjuncts were brandy and water, and, as soon as the appetite could take it, solid food well spiced. What seems still more remarkable, when one considers the habits and mode of living of the natives, is, that the same treatment proved equally successful with them. The native boat called "The Dingy," employed to convey messages, persons, etc., to and from the shore to the ship, frequently brought alongside, friends or relations of the boatmen, for the doctor's advice and medicines. To these Mr. Stedman gave his never-failing powders. Some of them he saw no more of, nor could he ascertain that any one of them died. On the contrary, the boatmen continued to bring more patients, or begged for powders for those who were too ill to be brought to the ship. To show his thankfulness and gratitude for the benefits conferred on his friends, the boat-master brought to Mr. Stedman, as a present, a string of beads, such, as he said, were worn only by Brahmins of high caste.

